# Illinois Veterinary Medical and Surgical Association

**Published:** 1900-09

**Authors:** W. A. Swain

**Affiliations:** Secretary


### ILLINOIS VETERINARY MEDICAL AND SURGICAL ASSOCIATION.

The eleventh semi-annual meeting, at Mattoon, Ill., August 8th, was called to order by President Dr. V. G. Hunt. Roll-call and reading of minutes of previous meeting.

President Hunt then delivered a masterly and touching address to those present, receiving much applause from the members.

By invitation of President Hunt, Dr. Martin, of Kankakee, then gave the Association a talk on "Spavin," defining the location and characteristics of this disease, its causes, and the probable outcome, giving his treatment.

Dr. Travis next read a paper on "Laryngitis," giving a careful account of the causes, symptoms, and results of this disease, and stating his mode of treatment. Responded to by Dr. V. G. Hunt. (Discussion among the members regarding the efficiency of tracheotomy in the treatment of laryngitis.) Dr. S. H. Swain, Dr. Martin, and Dr. John Osborne also responded.

Dr. S H. Swain was next on the programme for a paper on "Omphalitis," giving carefully its characteristics, causes and symptoms—also its hygienic and medical treatment. Responded to by Dr. G. G. Baumgartner. Dr. V.

G. Hunt gave a history of a case in his practice. Dr. N. P. Whitmore, of Gardner, responded.

Discussion on the efficiency of the present law regulating the practice of Veterinary Medicine and Surgery in the State of Illinois and the possibility of an amendment. This subject was very generally and warmly discussed by all present.

"Contagious Abortion" being next, by Dr. John Osborne. Dr. Osborne having a severe cold, his paper was read to the Society by the Secretary. This paper was responded to by Dr. Martin, of Kankakee and Dr. V. G. Hunt, and discussed generally by the Society.

Dr. V. G. Hunt followed next with an excellent paper on "Enteritis," giving graphically his treatment and the probable outcome.

Dr. S. H. Swain recited the history of a case of "Tetanus," giving his experience with the carbolic-acid treatment.

On motion the meeting was then adjourned until 8 A. M., August 9, 1900.

THURSDAY, AUGUST 9, 1900.

The applications for membership of Dr. Marsh, of Illiopolis, Dr. T. D. Skeen, of Garland, Ill., and Dr. N. P. Whitmore, of Gardner, Ill., were then read by the Secretary, and applications referred to the Committee on Membership, who examined into the proficiency and eligibility of the candidates.

The Committee on Membership reported favorably on the application of J. W. Marsh, of Illiopolis and N. P. Whitmore, of Gardner. The application of T. D. Skeen, of Garland, Ill., was tabled until the next meeting for lack of evidence.

On motion, the report of the committee was accepted. On motion, the Secretary cast the vote of the Association. Dr. Hunt then introduced the new members to the Association and the Association extended the right hand of fellowship to the new members.

On motion, the location, date, and place of next meeting were fixed in Decatur, Ill., at the Brunswick Hotel, on Wednesday and Thursday, January 10 and 11, 1901.

It was moved and seconded that the Chair appoint a committee to draft a set of resolutions censuring the Board of Live-stock Commissioners and Veterinary Examiners for the wholesale manner in which they have granted licenses to practice in the State of Illinois. Dr. N. P. Whitmore, Dr. S. H. Swain, and Dr. S. D. Brown were appointed on this committee. The committee reported the following resolutions:

Your Committee on Resolutions of Censure of the Board of Live-stock Commissioners and the Board of Veterinary Examiners of the State of Illinois, beg to report the following:

*Whereas*, a law has been passed known as the Veterinary Practice Act; and

*Whereas*, the law made it obligatory on the part of the Live-stock Commission to appoint a State Board of Veterinary Examiners to inquire into the qualifications of applicants for license to practice veterinary medicine and surgery; and

*Whereas*, M. H. McKillip, James Robertson, and Chas. H. Merrick were appointed as such Board of Veterinary Examiners; and

*Whereas*, the law clearly defines the duties of the Board of Veterinary

Examiners as to the examination of applicants not entitled to practice by reason of not being in possession of a diploma, or of not having been in practice three consecutive years in the State of Illinois; and

*Whereas,* the law requires that such applicants shall be examined in the following subjects, to-wit: veterinary anatomy, surgery, practice of medicine, obstetrics, pathology, chemistry, veterinary diagnosis, materia medica, therapeutics, physiology, sanitary medicine, and meat- and milk-inspection, and such other branches as the Board may prescribe; and

*Whereas,* it is well known by the members of this Association that the Veterinary Board have entirely ignored the law in making examinations, and have issued license to many persons of bad moral character, and who were entirely incompetent to practice, having neither practical nor literary knowledge of the science of veterinary medicine and surgery, thereby enabling such incompetent persons to point to the examination prescribed by law and which they are supposed to have passed; and

*Whereas,* many such applicants of bad moral character, and who were known to be incompetent to practice, have been protected by men of good reputation and of known truth and veracity; and

*Whereas,* said Board have overridden said protests, and have knowingly violated the letter and spirit of the law, and have made the license fee the only qualification upon which to issue a license to practice; therefore,

*Resolved,* that we, the members of the Illinois Veterinary Medical and Surgical Association, do condemn, censure, and deplore the conduct of said Board of Veterinary Examiners in the violation of their duties under the law, and further request their immediate removal from office.

S. H. Swain, V.S.,
N. P. Whitmore, M.D.,
S. D. Brown,
Committee.

The treatment of "Dysentery" was then discussed by the members, and the meeting was adjourned until January 10 and 11, 1901.

W. A. Swain,
Secretary.

---

The ninth annual meeting of the Missouri Veterinary Medical Association will be held at St. Louis, October 3d and 4th, and all veterinarians whether members of the Association or not, are cordially invited and urged to attend this meeting. Programme as follows: October 3d, "Surgical, Clinic, Consisting of the More Difficult Operations by the Latest Methods of Operating." Operators: Drs. R. C. Moore, Kansas City; F. W. O'Brien, Hannibal, and W. E. Martin, Perry, Mo. Papers, "Acute Bowel Trouble," by Dr. H. Bradley, Windsor; "Needed Veterinary Legislation in Missouri," Dr. Charles Ellis, St. Louis; "Influenza," Dr. E. Brainard, Memphis; "Canine Distemper," Dr. T. J. Menestrina, St. Louis; (subject not given), Dr. C. W. Crowley, St. Louis; "Parturient Paresis in the Cow," Dr. J. W. Connaway. Reports of cases. In the evening visit to St. Louis Exposition, entertainment by St. Louis veterinarians. October 4th, papers, "Infectious Ulcers of Vulva of Cattle," by Dr. C. Miller, St. Louis; "A Bacillus from an Infectious Vulva Disease of Cattle," Dr. J. W. Parker,

Kansas City; (subject not given), Dr. D. F. Luckey, Columbia; a report of the last meeting of the A. V. M. A., by its secretary, Dr. S. Stewart Kansas City; "Laminitis," Dr. L. M. Klutts, Clinton; "My Experience with Schmidt's Treatment of Parturient Apoplexy," Dr. F. W. O'Brien; (subject not given), Dr. T. E. White, Sedalia. Reports of the use f black-leg vaccine. Afternoon, a visit to the St. Louis Fair, under the direction of the veterinarians of St. Louis. Pathological exhibit.

THE twenty-eighth annual meeting of the American Public Health Association will be held at Indianapolis, Indiana, beginning October 22d, and continuing until October 27, 1900.

